# Nutritional functions of the funiculus in *Brassica napus* seed maturation revealed by transcriptome and dynamic metabolite profile analyses

**DOI:** 10.1007/s11103-016-0530-3

**Published:** 2016-08-18

**Authors:** Helin Tan, Xiaoe Xiang, Jie Tang, Xingchun Wang

**Affiliations:** 1 State Key Laboratory of Crop Genetics and Germplasm Enhancement, Nanjing Agricultural University, Nanjing, 210095 China; 2 Animal Sciences National Teaching Demonstration Center, Nanjing Agricultural University, Nanjing, 210095 China; 3 Crops Institute of Jiangxi Academy of Agricultural Sciences, Nanchang, 330200 China; 4 College of Life Sciences, Shanxi Agricultural University, Taigu, 030801 China; 5 Institute of Agricultural Bioengineering, Shanxi Agricultural University, Taigu, 030801 China

**Keywords:** *Brassica napus*, Funiculus, Nutrient transport, Metabolite profile, Transcriptome

## Abstract

The funiculus provides the sole channel of communication between the seed and the parent plant; however, little is known about its role in nutrient supply during seed maturation. Here, we investigated the dynamic metabolite profiles of the funiculus during seed maturation in *Brassica napus*. The funiculus was fully developed at 21 days after flowering (DAF), but the levels of nutrients, including carbohydrates, fatty acids, and amino acids, increased rapidly from 21 to 35 DAF. Orthogonal partial least squares discriminant analysis and correlation analysis identified 37 metabolites that correlated closely with seed fresh weight. To determine the influence of silique wall photosynthesis on the metabolites in the funiculus, we also covered the siliques of intact plants with aluminum foil; in these plants, the funiculus and silique wall had lower metabolite levels, compared with control. RNA-sequencing analysis of the funiculi in the dark-treated and light-exposed siliques showed that the expression of genes encoding nutrient transporters significantly increased in the funiculi in the dark-treated siliques. Furthermore, the transcripts encoding primary metabolic enzymes for amino acid synthesis, fatty acid synthesis and triacylglycerol assembly, and sucrose-starch metabolism, were also markedly up-regulated, despite the decline in metabolite levels of funiculi in the dark-treated silique. These results provide new insights into funiculus function in seed growth and synthesis of storage reserves in seeds, at the metabolic and transcriptional levels. The identification of these metabolites and genes also provides useful information for creating genetically enhanced oilseed crops with improved seed properties.

**Electronic supplementary material:**

The online version of this article (doi:10.1007/s11103-016-0530-3) contains supplementary material, which is available to authorized users.

## Introduction

Successful seed germination and seedling establishment are critical, challenging phases in the plant life cycle; to succeed in this stage, seeds mobilize their stored reserves until their own photosynthesis can support seedling growth (Tan et al. 2011). The storage reserves found in most plant seeds consist of carbohydrates, oils, and proteins, which constitute up to 90 % or more of the dry weight of the seed (Ruuska et al. 2002). In most field crops, these components also determine the economic value of the crop and provide the major value of seeds to humans and other animals. Therefore, plant breeders have focused substantial long-term efforts on enhancing the quantity and the quality of seed storage reserves (Ruuska et al. 2002; Agrawal and Thelen 2006).

Extensive studies on seed development have established that the components of the seed storage reserve begin to accumulate during the phase of seed development referred to as seed filling; the relative levels of proteins, lipids, and carbohydrates in the mature seed are also determined during seed filling (Murphy and Cummins 1989; Baud et al. 2002; Agrawal and Thelen 2006). Most seeds contain chloroplasts during the seed filling stage, but the seed predominantly acts as a sink tissue, importing most of its nutrients from source tissues to synthesize the storage reserves (Tan et al. 2011, 2015). The developing seed imports materials including organic nutrients, sucrose, amino acids, and potassium to support growth, especially during seed maturation. These nutrients move from the maternal plant via the vascular tissues in the center of the funiculus, to the seed coat, which further distributes the nutrients to other parts of the seed (Riens et al. 1991; Lam et al. 1995; Millar et al. 2015).

The funiculus anchors the developing seed to the placenta within the inner dorsal pod strands of the silique wall and also provides the only direct route for the transport of nutrients during seed development (Chan and Belmonte 2013). Morphological and histological observations showed that development of the funiculus occurs dynamically with seed development. Indeed, changes in the structural organization of the funiculus are necessary for normal seed growth, especially for its role in transport of nutrients into the seed (Mawson et al. 1994; Chan and Belmonte 2013; Khan et al. 2015; Millar et al. 2015). From the ovule to the globular stage of seed development, the vascular strand (particularly the phloem) becomes much larger by increasing the number of cells of the vasculature. This facilitates the transport of metabolites to the seed (Chan and Belmonte 2013).

Comprehensive molecular analysis of the distal seed coat (SC) and the chalazal seed coat (CZSC) of *Arabidopsis thaliana* and *Brassica napus* revealed that specific populations of mRNAs accumulated specifically in the CZSC and not in other seed regions (Belmonte et al. 2013; Khan et al. 2015; Millar et al. 2015). A systems biology approach that compared the transcriptome of the funiculus to the transcriptomes of the SC and CZSC in both space and time revealed profound differences in the molecular machinery controlling adjacent seed coat sub-regions at the transcriptional level. This comparison showed that transcripts involved in the transport and metabolism of sugar, amino acids, lipids, and hormonal regulation are expressed in the funiculus at specific stages of seed development that coincide with the timing of integral processes associated with embryonic growth and the accumulation of oil and protein (Khan et al. 2015). These results provide a putative molecular basis for understanding the development of the funiculus; however, these activated genes involved in various metabolic pathways remain poorly understood, although they likely have important functions in supply and transport of nutrients demanded by seed filling, thereby controlling the quantity and the quality of seed storage reserves. Hence, understanding the dynamics of the metabolite features, as well as the related metabolic genes, can help in comprehensive elucidation of the pivotal role of the funiculus in regulating nutritive storage in the seed.

Here, we profiled the dynamic metabolome of the funiculus during the biosynthesis of storage reserves in *B. napus* seeds. We identified metabolites associated with seed weight and showed that some of the metabolites were altered in dark-treated siliques. In addition, we determined the expression profiles of candidate genes involved in metabolite transportation and metabolic pathways in the funiculus using RNA-sequencing. This combination of metabolomic and transcriptomic analysis enhances our understanding of the function of the funiculus during seed maturation.

## Materials and methods

### Plant growth and sample collection

Plants of the oilseed rape (*B. napus*) cultivar Zhongshuang 11 were sown in late September 2013 and grown under field conditions with a seedling density of 20 × 40 cm. To investigate the dynamic metabolite profile of the funiculus during seed maturation, the flowering days of 30 plants were noted. The siliques were collected for statistical analysis of silique fresh weight, silique length, seed fresh weight, and funicular length every week until 35 days after flowering (DAF). At 21, 28, and 35 DAF, siliques were harvested from the plants and chilled on ice for up to 2 h prior to funiculus collection. Funiculi were carefully removed from the siliques and rapidly frozen in liquid nitrogen, and then stored in −80 °C until they were used for extraction of metabolites. To determine the effects of metabolite levels in the silique wall on those in the funiculus, half of each silique of 30 plants was shaded with aluminum foil at 21 DAF. After 4 and 7 days of shade treatment (i.e., at 25 and 28 DAF), siliques were collected and the funiculi were carefully gathered from the silique walls of the shaded portion and non-shaded portion, respectively, and then stored at −80 °C until use. Silique walls and funiculi were separated for analysis as described below. Three to six biological replicates were analyzed, with each replicate consisting of five plant samples.

### Metabolite extraction

Metabolite extraction was performed as previously described with minor modifications (Tan et al. 2015). Briefly, 100 mg of sample stored at −80 °C was ground in liquid nitrogen and transferred to 10 mL centrifuge tubes. Pre-cooled (−40 °C) pure methanol (1.4 mL) was added and the samples were then vortexed for 10 s, followed by the addition of 60 µL adonitol (0.2 mg/mL stock in dH_2_O) as an internal quantitative standard. The mixed samples were vortexed again for 10 s. Tubes were placed into an ultrasound machine at 70 °C for 30 min, and then 1.4 mL ddH_2_O (4 °C) was added and the samples were vortexed for 1 min, followed by centrifugation at 11,000*g* for 10 min. Then, 1 mL supernatant was transferred into a new microfuge tube and dried under a moderate stream of nitrogen. The dried samples were dissolved in methoxyamine pyridine (60 µL of a 15 mg/mL solution) and vortexed for 30 s, and then incubated for 90 min at 37 °C. Lastly, 60 µL of *N*-methyl-*N*-(trimethylsilyl) trifluoroacetamide (MSTFA) reagent (containing 1 % trimethylchlorosilane, TMCS) was added and the mixture was incubated for 150 min at 37 °C.

### Metabolite analysis

Metabolites extracted from samples were analyzed as previously described with minor modifications (Tan et al. 2015). Briefly, the extracted metabolites were analyzed using an Agilent 7890A GC System coupled to an Agilent 7890A-5975C Inert XL EI/CI Mass Spectrometric Detector (MSD) System (Agilent Technologies, Santa Clara, CA, USA). Gas chromatography was performed on a DB-5 Capillary Column (30 m × 0.25 mm × 0.25 µm, Agilent J & W Scientific, Folsom, CA, USA) to separate the derivatives. The injection temperature was 280 °C, the interface was set to 150 °C, and the ion source was adjusted to 230 °C. The temperature gradient program was as follows: initial temperature of 60 °C for 1 min, +10 °C/min up to 300 °C, and a hold at 300 °C for 15 min. Mass spectrometry was determined by the full-scan method ranging from 33 to 600 (m/z). A series of n-alkanes (C7–C40) and blank control samples was analyzed along with samples to calculate retention indices (Schauer et al. 2005).

### Data processing and metabolite identification

Raw GC/MS data were converted into the CDF format (NetCDF) using Agilent GC/MS 5975 data analysis software and were subsequently processed with XCMS (http://www.bioconductor.org) using XdCMS default settings with the following changes: xcmsSet (fwhm = 3, snthresh = 3, max = 300, mzdiff = 0.5, step = 0.1, steps = 2), rector (method = “linear”, family = “gaussian”, plottype = “mdevden”), and bandwidth (bw) of five. The identities of the compounds responsible for specific resolved peaks in the mass spectra were determined by performing database searches based on the mass spectra and the compounds’ chromatographic retention indices. The databases used were the NIST 2011 Mass Spectra Library, an in-house database established by Ume a Plant Science Centre (UPSC), and the mass spectra library maintained by the Max Planck Institute in Golm, Germany (http://csbdb.mpimp-golm.mpg.de/csbdb/gmd/gmd.html).

### Metabolite data analysis

Each metabolite was expressed as a peak area normalized to the adonitol internal standard. For multivariate statistical analysis, the XCMS output was further processed using Microsoft Excel (Microsoft, Redmond, WA, USA). Finally, normalized data were imported into Simca-P software (version 14.0, http://www.umetrics.com/simca) for multivariate statistical analyses, including principal component analysis (PCA) and partial least squares discriminant analysis (PLS-DA). All data were mean-centered and Pareto-scaled prior to PCA and PLS-DA. Discriminating metabolites were identified using a statistically significant threshold of variable influence on projection (variable influence on projection values, VIP > 1.0) values obtained from the PLS-DA model and were further validated by *t* test analysis. Metabolites with VIP values of greater than 1.0 and p values of below 0.01 (threshold) were selected as discriminating metabolites between two classes of samples. Heat maps and expression lines prepared with the TIGR MEV 4.9 software package (Saeed et al. 2003) were used to visualize metabolite responses. The heat maps were generated based on the average measured relative abundance of individual metabolites in three to six biological replicates. The correlation analysis was completed with SPSS (Statistical Product and Service Solutions, SPSS Inc.) software (Green and Salkind 2010).

### RNA extraction, library preparation, and sequencing

Total RNA was prepared from 100 mg of funicular tissue using TRIzol Reagent (Sigma–Aldrich, Dorset, UK). Tissue samples were homogenized in 1 mL of TRIzol reagent and 300 μL chloroform and subsequently precipitated using 500 μL isopropanol (Sigma Chemical, Wicklow, Ireland). RNA samples were stored at −80 °C. Then, 20 μg of total RNA from each sample was treated with RNase-free DNase (QIAGEN, Crawley, West Sussex, UK) to prevent genomic DNA contamination and purified using the RNeasy Mini Kit in accordance with the manufacturer’s instructions (QIAGEN, Crawley, West Sussex, UK). RNA quality and quantity were assessed using automated capillary gel electrophoresis on a Bioanalyzer 2100 with RNA 6000 Nano Labchips, according to the manufacturer’s instructions (Agilent Technologies Ireland, Dublin, Ireland). Then, 5 μg of RNA from each sample was used for library construction using standard protocols. Paired-end libraries were constructed for control funiculi at 28 DAF (CF28) and the funiculi associated with silique walls covered with aluminum foil after 7 days treatment, namely 28 DAF (SF28). The median insert size was 250 bp. Two biological replicate RNA samples from each funiculus were sequenced using the Illumina HiSeq 2500V4 system. The total number of mate-paired reads for each sample ranged from 8,000,000 to 13,000,000. Read lengths of 125 bp were collected.

### Read alignment and data analysis

The raw reads were first cleaned with the NGSQCToolkit_v2.3, and then the cleaned reads were aligned to the *B. napus* genome v4.1 (Chalhoub et al. 2014) using the ultrafast Bowtie aligner (Langmead et al. 2009). The alignment allowed for only one mismatch per read. Reads that hit more than one locus were filtered from the Bowtie output and only reads that matched a single locus were used for further analysis. Read counts were obtained from the Bowtie output using a set of Perl scripts. The expression level of genes was calculated as RPKM (reads per kb per million reads) (Mortazavi et al. 2008). Differential expression of these transcripts between CF28 and SF28 was then estimated using Cuffdiff (Trapnell et al. 2010). Metabolic pathway analysis was performed using Mapman software (Thimm et al. 2004).

## Results

### Characterization of silique and funiculus growth during seed development

**Fig. 1:**
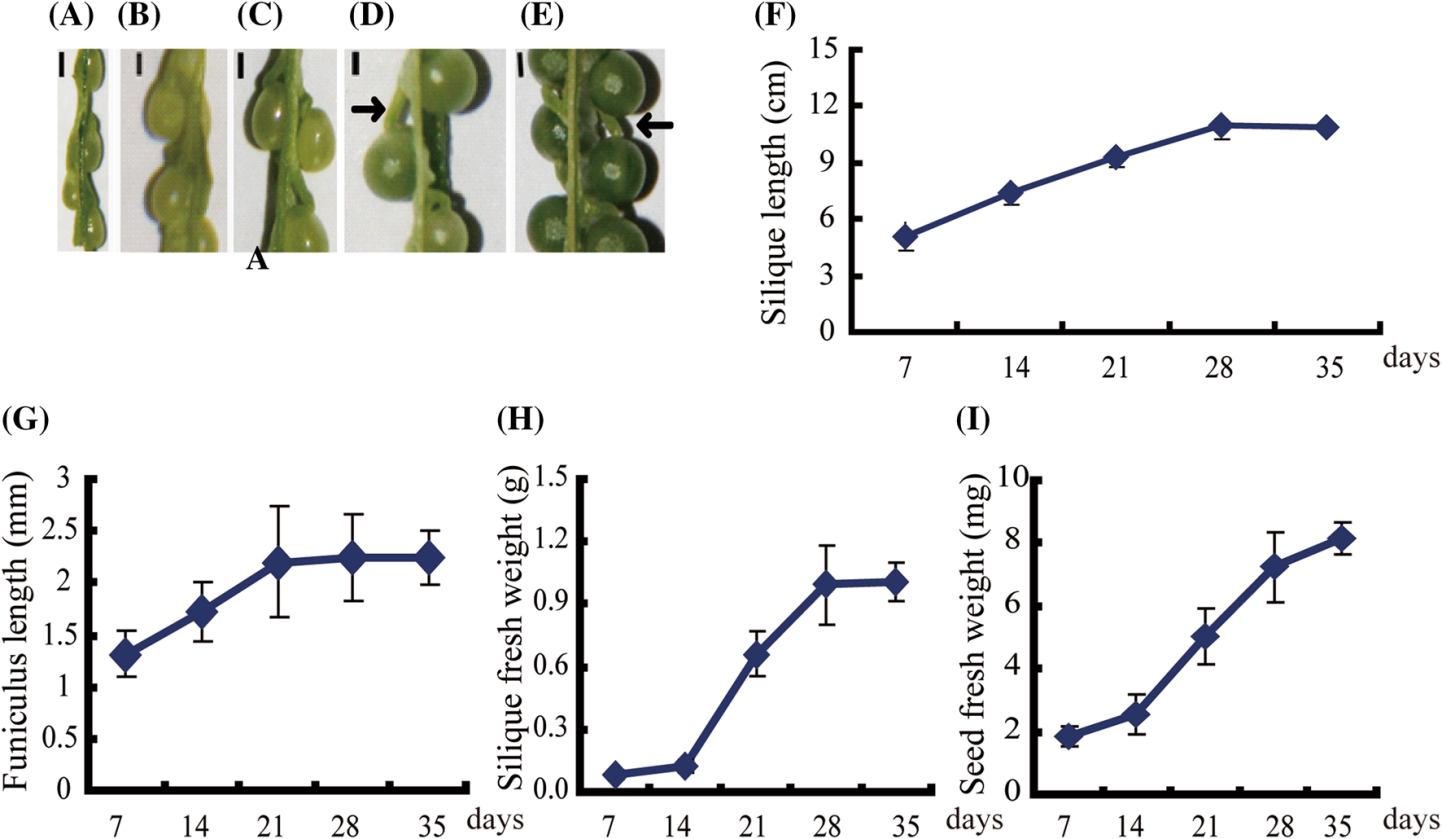
Characterization of the developing silique, funiculus, and seed. **a–e** Funiculi and seeds in siliques collected at 7, 14, 21, 28, and 35 DAF, respectively. *Bars* 1 mm and the *row* indicates the funiculus. **f** Mean lengths and standard deviations of 20 siliques at the indicated time points. **g** The mean lengths of 30 funiculi from 10 siliques at different developmental stages. *Bars* indicate standard deviations. **h** The mean fresh weights and standard deviations of siliques (three biological replicates, each with 10 siliques). **i** The mean fresh weights and standard deviations of seeds at different developmental stages (three biological replicates, each replicate with 10 siliques)

In *B. napus*, developing seeds in the silique are anchored to the replum (placenta) via the funiculus (Fig. 1a–e). From 7 to 14 days after flowering (DAF), the young seeds are transparent (Fig. 1a, b) and from 21 DAF onwards, the seeds appear green due to synthesis of chlorophyll in the embryo. The green embryo rapidly enlarges and fills the seed by 35 DAF (Fig. 1c, e). To accommodate the enlarged seeds, the siliques rapidly elongate from 7 to 28 DAF and stop elongating by 35 DAF (Fig. 1f). Concomitantly, the funiculus increases in length from 7 to 21 DAF, but not between 21 and 35 DAF (Fig. 1g). The increase in silique wall length is accompanied by a rapid increase in fresh weight between 7 and 28 DAF, and a slower increase between 28 and 35 DAF (Fig. 1h). By contrast, the seed fresh weight increased consistently during these developmental stages (Fig. 1i), with the weight increasing by 143.94 % between 21 and 28 DAF and 112.43 % between 28 and 35 DAF. This period is the key phase when the embryo absorbs massive amounts of nutrients to synthesize the storage reserve.

### Dynamic metabolite profiles of the funiculus during seed maturation

**Fig. 2:**
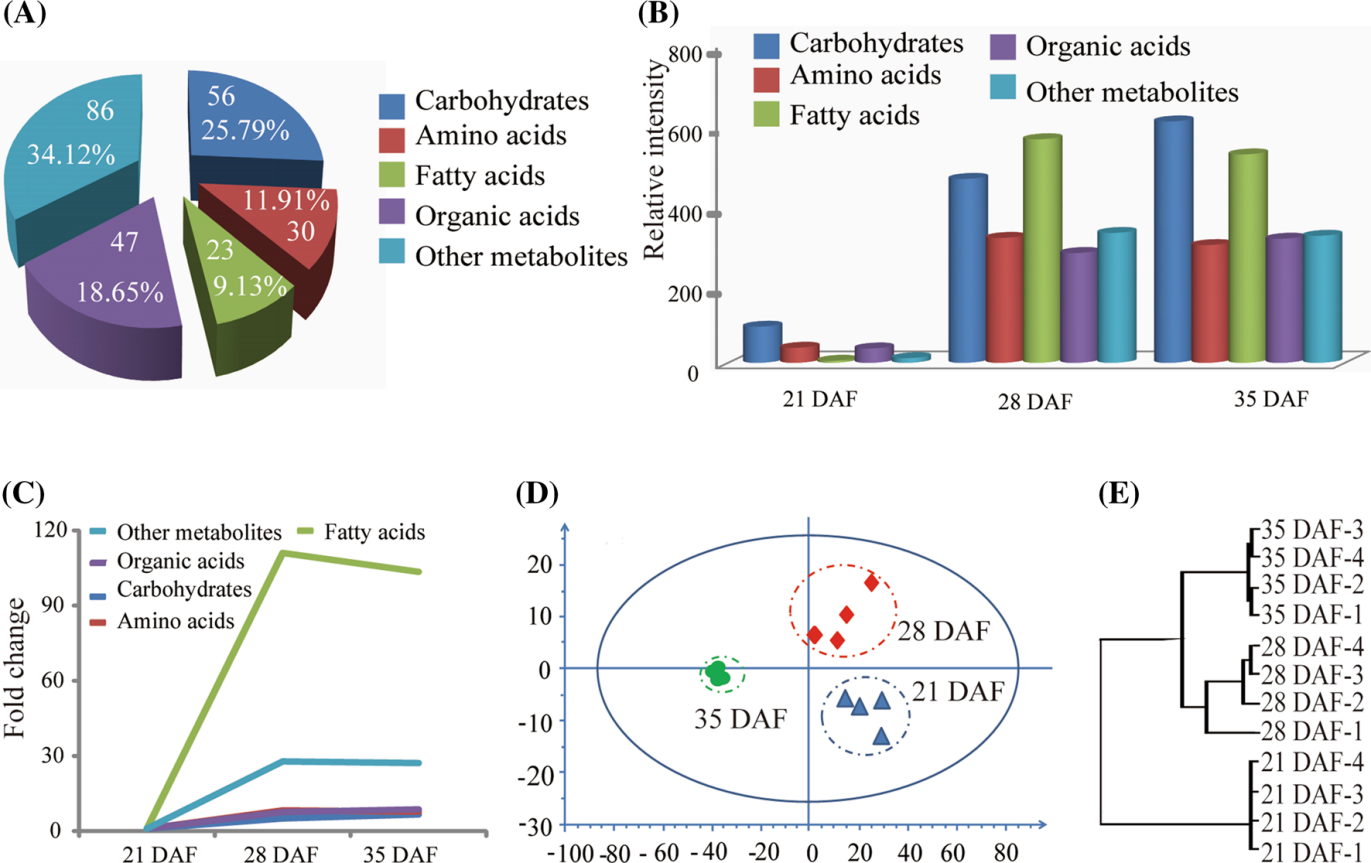
Dynamic metabolite profiles of the funiculus during seed maturation. **a** Distribution of metabolites in the funiculus. **b** Relative levels of metabolites in the funiculus at different time points. **c** Fold changes of the relative levels of metabolites at different time points. **d** PCA of funiculus metabolite profiles with all identified metabolites at three time points by SMICA (V 14, p < 0.05). **e** Hierarchical clustering of funiculi metabolite profiles based on all identified metabolite levels; four biological replicates were performed for each time point

The rapid increase in seed fresh weight requires import of a large amount of nutrients via the funiculus. To investigate the role of this nutrient import in *B. napus* seed filling, we used GC-MS to identify compounds in the funiculus at 21, 28, and 35 DAF, which represent key stages of seed filling in *B. napus* (Vigeolas et al. 2003). We identified 251 metabolites with known structures, including amino acids and their derivatives (30), carbohydrates (56), organic acids (47), fatty acids and lipids (23), and other complex metabolites (86) (Supplemental Table S1; Fig. 2a). Among these metabolites, the total relative content of carbohydrates and organic acids increased from 21 to 35 DAF (Fig. 2b), with the increase being greater between 21 and 28 DAF (5–8 fold relative to levels at 21 DAF) than between 28 and 35 DAF (1–2 fold) (Fig. 2c); also, the relative contents of other complex metabolites increased rapidly between 21 and 28 DAF and then to a lesser extent between 28 and 35 DAF (Fig. 2b), however, the total relative contents of fatty acids and lipids increased more rapidly than those of other metabolites, with a 111-fold increase at 28 DAF compared to 21 DAF, and a 103-fold increase between 21 and 35 DAF (Fig. 2b, c). The total relative content of primary metabolites, including carbohydrates, amino acids and their derivatives, and fatty acids and lipids displayed substantial changes and accounted for 68.87 % of the total identified metabolites at 28 and 35 DAF, while organic acids and other complex metabolites accounted for only 31.12 %.

Principal component analysis (PCA) of metabolite profiles at the three stages showed that these metabolite profiles were distinct (Fig. 2d) and the metabolite profiles at 21 DAF and 28 DAF were closer than the profile at 35 DAF, which indicated that metabolite contents in the funiculus contributed to the different metabolite profiles at these stages. Moreover, the metabolite contents increased from 21 DAF to 28 DAF and then decreased at 35 DAF (Fig. 2d), and the fold changes of metabolite contents, such as amino acids, fatty acids, and lipids, showed similar patterns (Fig. 2c). Further hierarchical clustering analysis showed that all of the metabolite profiles obviously clustered into three groups: 21 DAF group, 28 DAF group, and 35 DAF group, but the metabolite profiles at 28 and 35 DAF were more similar than those at 21 DAF (Fig. 2e), consistent with the observed content changes of primary metabolites at the three stages (Fig. 2b). These results showed that the funiculus possessed plentiful nutrients during seed filling and the proportions of these nutrients varied with the seed development stages.

### Metabolites correlated with seed increase

**Fig. 3:**
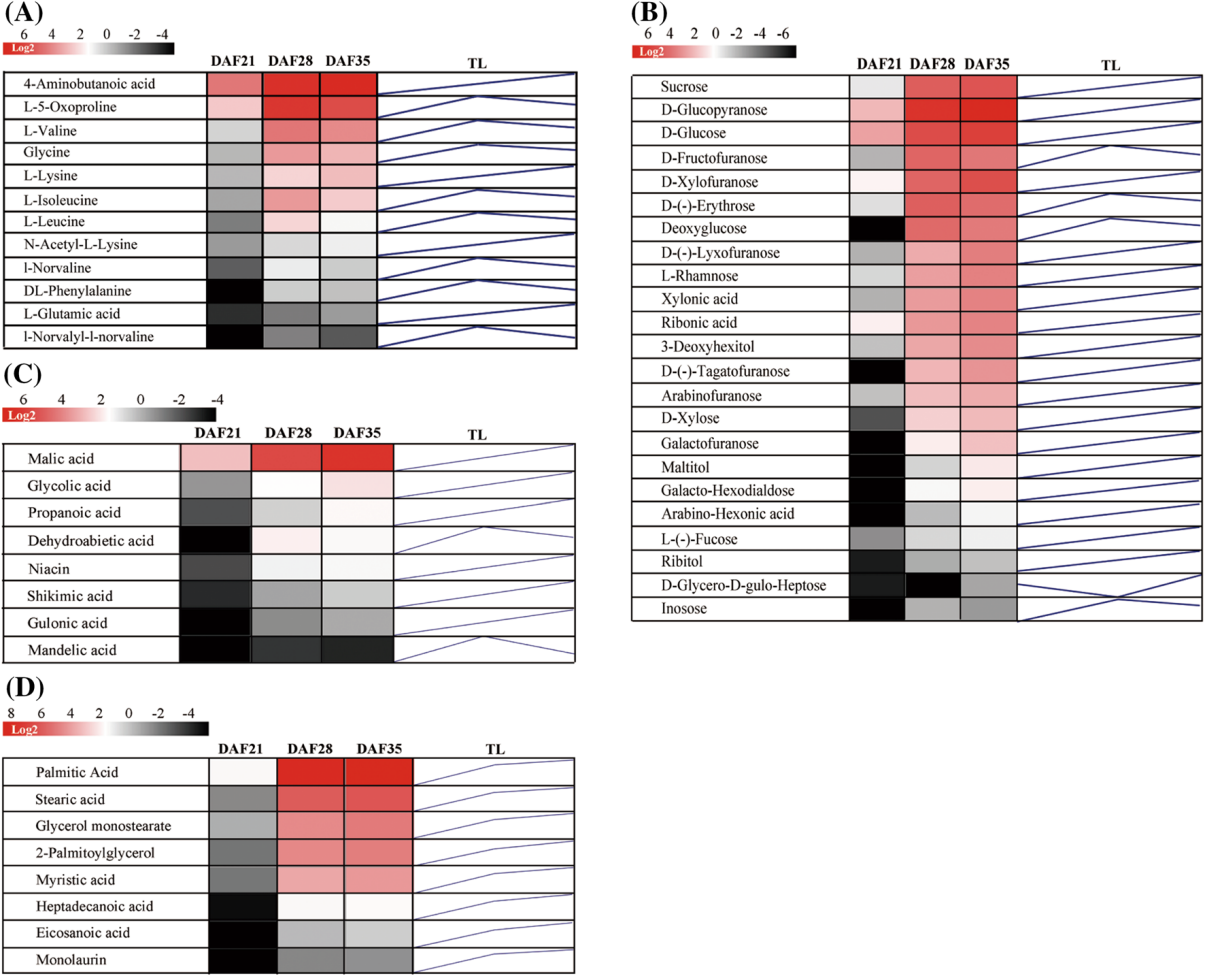
Changes in the abundance of metabolites in the funiculus during seed maturation. **a–d** Heat maps (*colored box*) and trend lines (TL) of different amino acids, carbohydrates, organic acids, fatty acids, and lipids at 21, 28, and 35 DAF, respectively. The *color change in each box* reflects the metabolite abundance, and the TL line was plotted with the metabolite levels in the funiculus at 21, 28, and 35 DAF. The metabolite levels consistently increased from 21 to 35 DAF whereas the metabolite level decreased between 28 and 35 DAF

To explore metabolites correlated with seed filling, particularly with the seed fresh weight, we first used orthogonal partial least squares discriminant analysis (OPLS-DA) of the metabolite profiles. This analysis indicated that the relative contents of 68 metabolites, including 12 amino acids and their derivatives, 23 carbohydrates, 8 fatty acids and lipids, 8 organic acids, and 17 other complex metabolites, differed significantly (variable influence on projection values, VIP > 1.0; *t* test <0.01) at 21, 28, and 35 DAF (Fig. 3).

These metabolites could be directly imported into developing seeds for filling, or indirectly imported into seeds after turnover by metabolic enzymes. To test the relationship of these metabolites, we conducted metabolite–metabolite correlation analysis of our data with SSPS; this revealed positive and negative correlations among metabolites at the three developmental stages, 21, 28, and 35 DAF. At a threshold correlation value of >0.50 (r value >0.5), we identified 50,523 pairs of metabolites with positive correlations and 4748 pairs with negative correlations. Among these metabolite–metabolite correlations, 3955 pair-wise associations were significant at p values of at least 0.05 (p < 0.05). The vast majority of the correlations were among same classes of metabolites such as carbohydrates, amino acids, and lipids.

We further performed correlation analysis between the funiculus metabolites and seed fresh weight at the three developmental stages. This analysis revealed that most of the metabolites in the funiculus positively correlated with seed fresh weight. Of these, 37 metabolites showed close correlations, with correlation coefficients r > 0.97 (Supplementary Table S2). However, linear regression analysis of the 38 metabolites showed that only sucrose and cyclopentene were directly dependent variables for seed fresh weigh with the standardized coefficients 0.368 and 0.634, respectively, in the predicted model.

### Shading the silique decreased metabolite contents in the silique wall and the funiculus

**Fig. 4:**
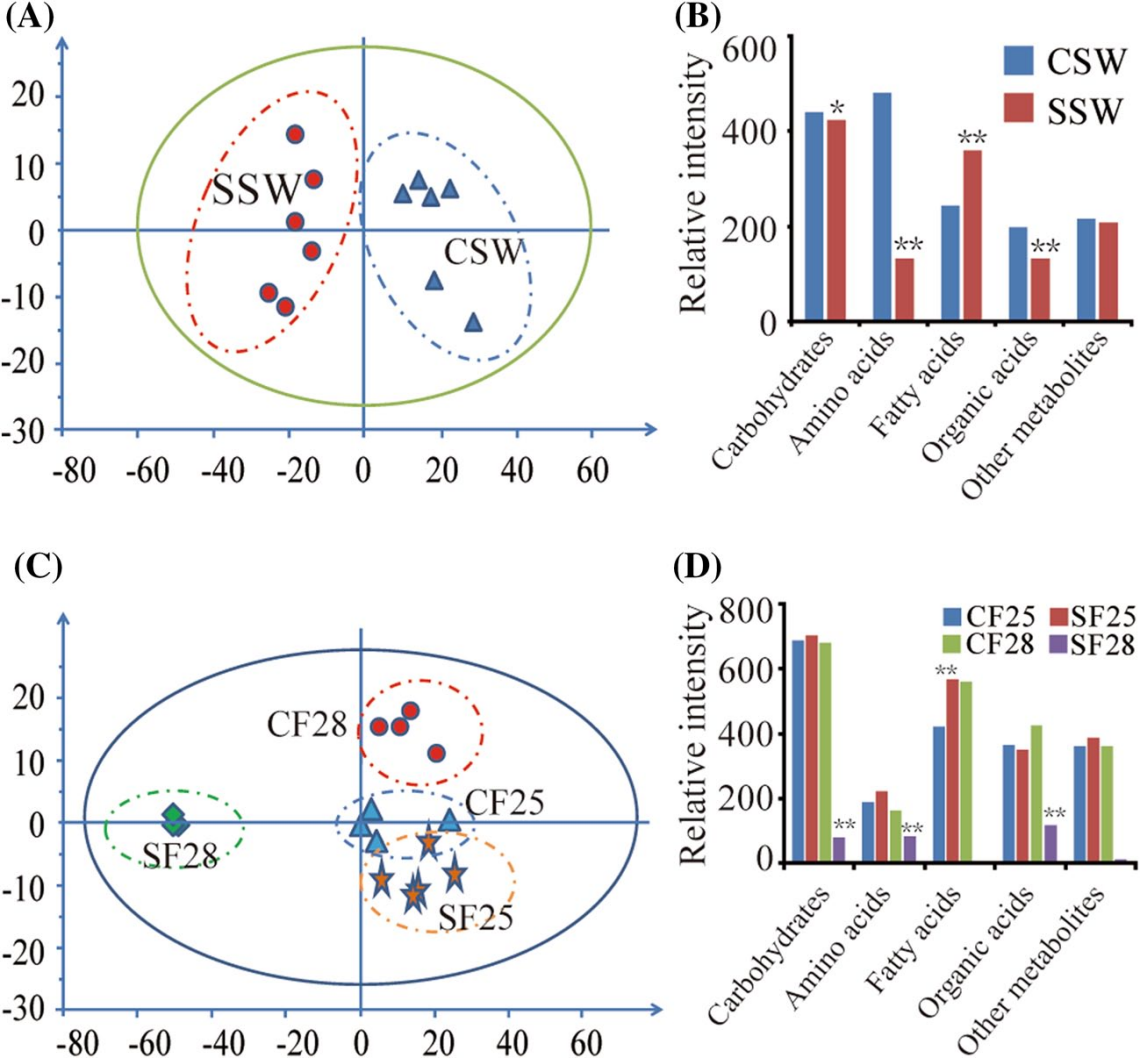
Differences in the metabolite profiles of funiculi associated with the shaded and non-shaded silique walls. The siliques were shaded with aluminum foil at 21 DAF, silique walls were collected at 28 DAF (CSW, SSW), and funiculi associated with the silique wall were collected at 25 and 28 DAF (CF25, CF28, SF25, and SF28). **a** PCA of control silique wall metabolite profiles (CSW) and shaded silique wall metabolite profiles (SSW) (p < 0.05). **b** Relative total levels of metabolites in CSW and SSW. **c** PCA of metabolite profiles of funiculi associated with the control silique wall at 25 (CF25) and 28 (CF28) DAF, and of funiculi associated with the shaded silique wall at 25 (SF25) and 28 (SF28) DAF (p < 0.05). **d** Relative total levels of metabolites in CF25, CF28, SF25 and SF28

The silique wall is the main photosynthetic tissue providing nutrients for the seed during seed filling (Fang et al. 2012; Tan et al. 2015). To unmask the relationship between metabolite abundances in the silique wall and funiculus, we shaded the silique at 21 DAF with aluminum foil to block silique photosynthesis. Subsequently, we collected the shaded and control silique walls at 28 DAF (i.e. after 7 days of dark treatment). Metabolites in the shaded-silique wall (SSW) and control silique wall (CSW) were analyzed by GC-MS. PCA analysis indicated that the levels of metabolites in the SSW changed significantly compared to the CSW (Fig. 4a). The levels of carbohydrates and other complex metabolites decreased slightly in SSW compared to CSW, but the levels of amino acids and their derivatives, and organic acids were reduced by 72.4 and 33.1 %, respectively (Fig. 4b). By contrast, the levels of fatty acids and lipids in SSW were up to 150 % greater than those in CSW (Fig. 4b), which is consistent with our previous results that fatty acid contents increased in dark-treated silique walls (Tan et al. 2015).

To test whether metabolite contents in the funiculus showed a corresponding decrease, we analyzed metabolites in funiculi of the shaded siliques after 4 and 7 days of dark treatment, corresponding to 25 and 28 DAF. We designated these funiculi as SF25 and SF28, and the corresponding control funiculi of the non-shaded siliques as CF25 and CF28. PCA analysis showed that the metabolite profile of SF28 was distinct from those in CF28, CF25, and SF25 (Fig. 4c). The contents of carbohydrates were 8.5-fold lower in SF28 than in CF28 (Fig. 4d); moreover, the amino acids and their derivatives, and levels of organic acids in SF28 decreased nearly twofold and fourfold, respectively, compared with CF28. The levels of fatty acids and lipids in SF28, by contrast, decreased 150-fold compared to CF28 (Fig. 4d). However, the relative total contents of carbohydrates in CF25, CF28, and SF25 were barely affected by shading (Fig. 4d). These results showed that shading the silique decreased not only the metabolite contents in the silique wall, but also in the funiculi, suggesting the nutrient levels in silique walls affect the levels in funiculi, and supporting the importance of photosynthesis in the silique wall for seed filling.

### Metabolite contents in silique walls directly affected the metabolite contents in funiculi

**Fig. 5:**
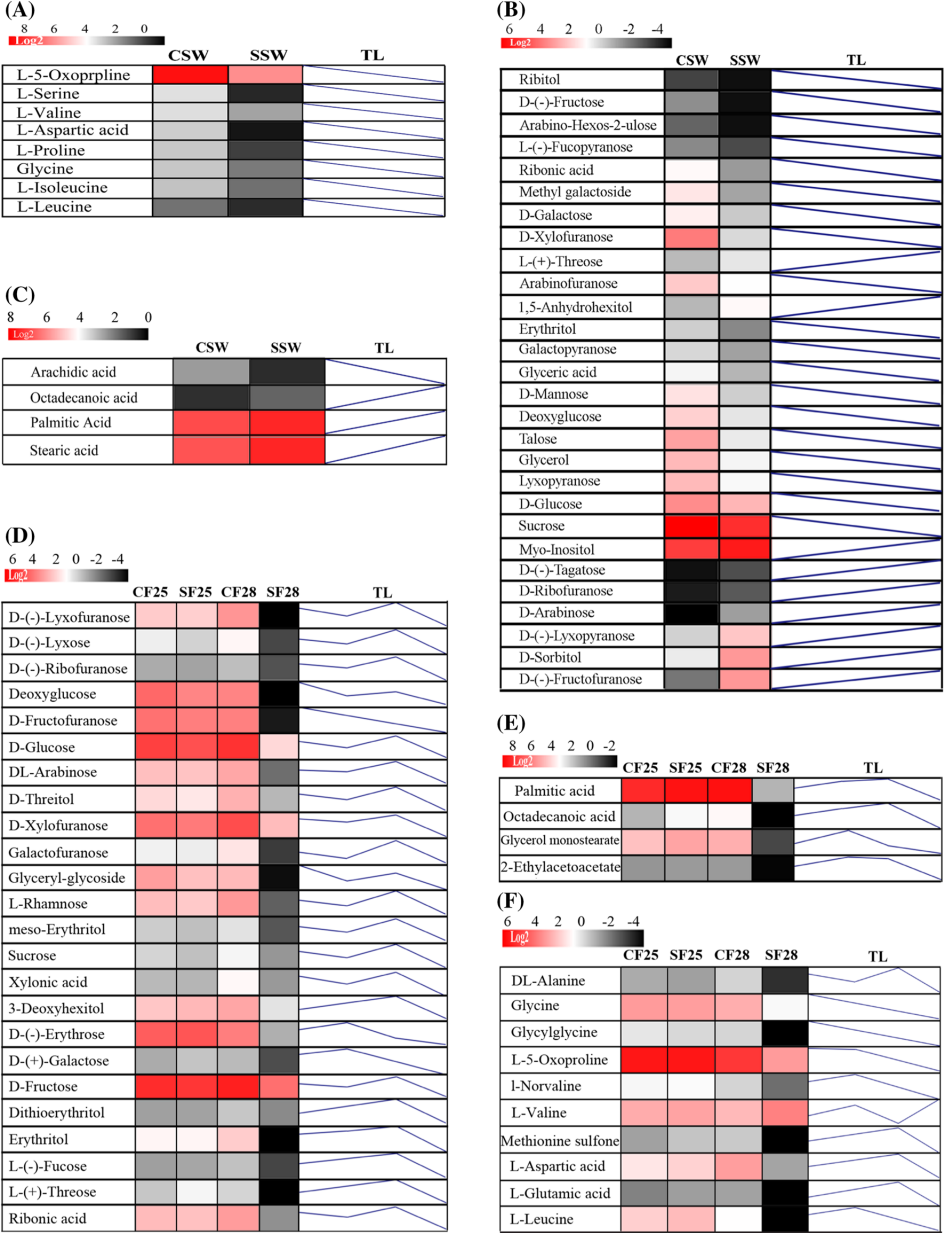
Silique shading altered the levels of metabolites in the funiculus and silique wall. **a–c** Heat maps (*color box*) and trend lines (TL) of amino acids, carbohydrates, fatty acids, and lipids in the walls of control (CSW) and shaded (SSW) siliques. The *color of each box* represents the level of the metabolite and the TL line shows variations in metabolite levels between CSW and SSW. **d–f** Heat maps (*colored boxes*) and trend lines (TL) of different carbohydrates, amino acids, fatty acids, and lipids in the funiculus associated with control (CF25, CF28) and shaded (SF25, SF28) silique walls. Funiculi were collected at 25 (CF25 and SF25) and 28 (SF28 and CF28) DAF. The *color of each box* represents the level of the metabolite, and the TL shows variations in metabolite levels amongst CF25, SF25, CF28, and SF28

To explore the changed metabolites, we compared the metabolite profiles of the CSW and SSW by OPLS-DA. Statistical analysis showed that 65 metabolites identified in SSW exhibited significant differences (VIP > 1.0; *t* < 0.01) compared to CSW, including 8 amino acids and their derivatives, 28 carbohydrates, 4 fatty acids and lipids, 14 organic acids, and 11 other complex metabolites. Next, we compared the metabolite profiles of the CF25, CF28, SF25, and SF28 and identified 57 significantly changed metabolites (VIP > 1.0; *t* < 0.01), including 10 amino acids and their derivatives, 24 carbohydrates, 4 fatty acids and lipids, 10 organic acids, and 9 other complex metabolites (Fig. 5a–c). Correlation analysis among the identified metabolites that differed between silique walls and funiculi found 661 pairs of positive correlations, and 205 pairs of negative correlations at a threshold of correlation value >0.50 (r value >0.5). Among these metabolite–metabolite correlations, 574 pair-wise associations significantly differed at p value at least 0.05 (p < 0.05). Further analysis found 22 significantly changed metabolites shared in SSW, SF25, and SF28, including 5 amino acids, 11 carbohydrates, 2 fatty acids, and 4 organic acids (Fig. 5d–f). Of these, the contents of eight metabolites increased in SSW compared with CSW, while only l-valine increased in SF28 compared with CF28. The contents of the other metabolites all decreased in silique wall and funiculus following shading treatment, suggested that the levels of these metabolites in the silique wall may directly affect their levels in the funiculus.

**Fig. 6:**
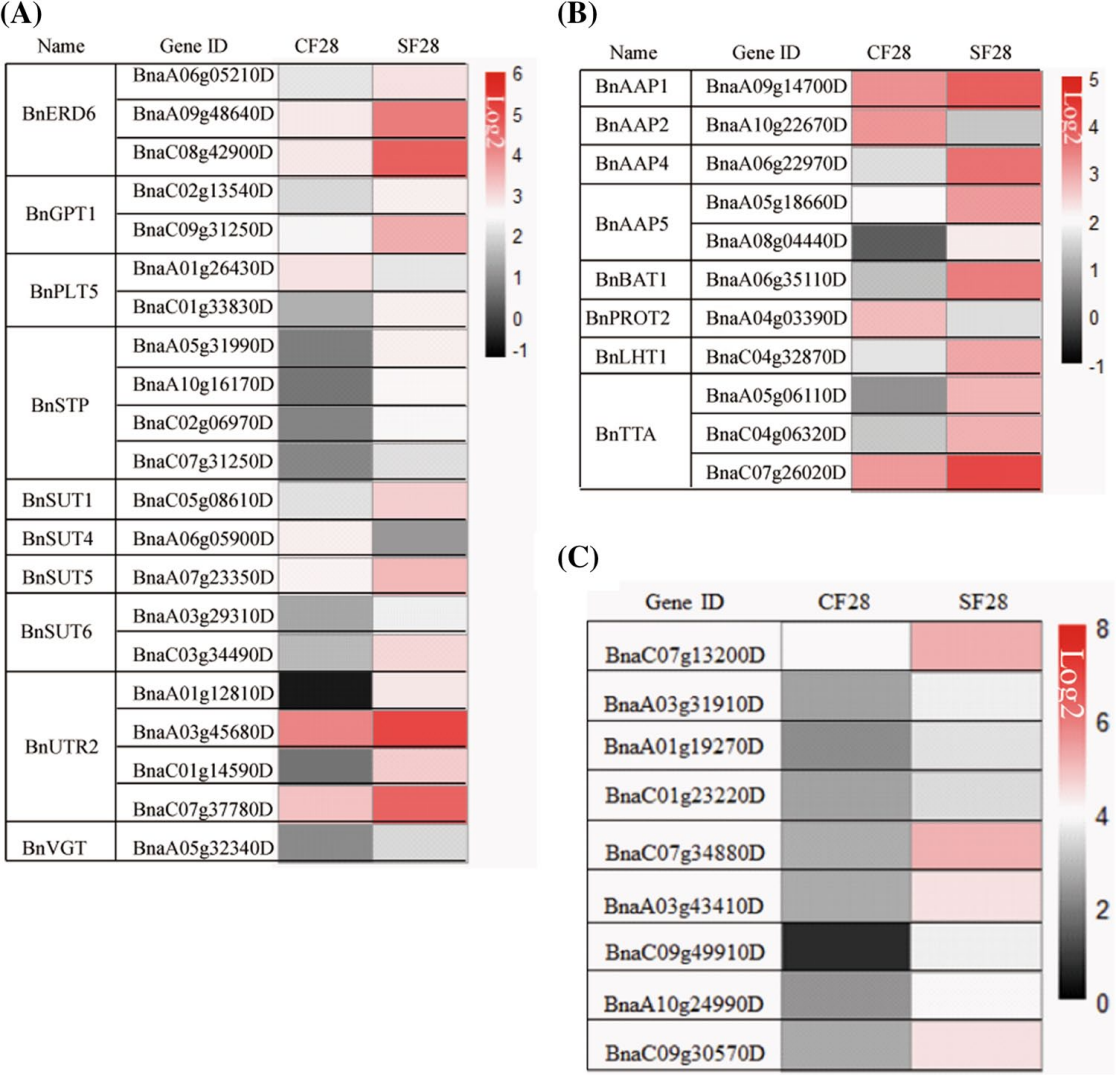
The expression of nutrient transport genes in CF28 and SF28. **a** The genes encoding sugar transport proteins. **b** The genes encoding amino acid transport proteins. **c** The genes encoding lipid transport proteins

To test whether the metabolite changes in the funiculus affected the expression of nutrient transport genes, we conducted transcriptome sequencing of SF28 and CF28 using the Illumina Hiseq 2500 V4. To predict candidate genes important for nutrient transport, we screened for genes encoding proteins involved in the transport of carbohydrates, amino acids, and lipids (Fig. 6a–c). *SUT* encodes a high-affinity transporter essential for phloem loading and long-distance transport of sucrose, the abundances of the transcripts encoding BnSUT1 (BnaC05g08610D), BnSUT5 (BnaA07g23350D), and BnSUT6 (BnaC03g34490D, BnaA03g29310D) were elevated up to threefold in SF28. The transcript abundances of *BnGTP1* (BnaC09g31250D, BnaC02g13540D) encoding glucose 6-phosphate/phosphate transporter 1, *BnPLT5* (BnaC01g33830D) encoding polyol/monosaccharide transporter 5 localized in the plasma membrane, and *BnUTR2* (BnaC01g14590D, BnaA03g45680D, BnaA01g12810D, BnaC07g37780D) encoding UDP-galactose transporter 2, were up-regulated by threefold in SF28. *BnPMT5* (BnaC01g33830D) encodes a plasma membrane-localized polyol/cyclitol/monosaccharide-H^+^-symporter, which catalyzes the energy-dependent membrane passage of numerous monosaccharides, including pyranose ring-forming and furanose ring-forming hexoses and pentoses, and its transcript levels significantly increased by threefold in SF28. Also, *BnVGT1* (BnaA05g32340D) encodes a vacuolar membrane-localized glucose transporter that can also transport fructose, and its transcript abundance increased by close to threefold in SF28. However, the transcript abundances of *BnSUT4* (BnaA06g05900D) and *BnPLT5* (BnaA01g26430D) were down-regulated by three and eightfold, respectively, and *BnPLT2* (BnaCnng67460D) was silenced in SF28 (Fig. 6a).

Amino acids constitute the main form of N transport and the abundances of the transcripts encoding the bidirectional amino acid transporter BnBAT1 (BnaA06g35110D), neutral amino acid transporter BnAAP1 (BnaA09g14700D), BnAAP4, (BnaA06g22970D), BnAAP5 (BnaA05g18660D, BnaA08g04440D), lysine histidine transporter BnLHT (BnaC04g32870D) and transmembrane amino acid transporter family protein (BnaA05g06110D, BnaC04g06320D, BnaC07g26020D) were elevated by over twofold in SF28 compared to CF28 (Fig. 6b). *BnAAP2* (BnaA10g22670D) and *BnPROT2* (BnaA04g03390D) encode proline transporters with affinity for gly betaine, proline, and GABA; their transcript abundances decreased three and sixfold in SF28 compared to CF28, respectively (Fig. 6b).

In SF28 compared to CF28, the abundances of nine transcripts increased to varying degrees; these transcripts (BnaC07g13200D, BnaA03g31910D, BnaA01g19270D, BnaC01g23220D, BnaC07g34880D, BnaA03g43410D, BnaC09g49910D, BnaA10g24990D, BnaC09g30570D) encode plant lipid transfer proteins (LTPs). Based on the complexity of the LTP gene families, a systematic classification is difficult, with no commonly used and accepted classification; therefore, here the nine predicted *LTP* genes were not classified (Fig. 6c).

All together, these results showed that the decreased metabolite contents in silique walls led directly to corresponding reductions in metabolite contents in the funiculus, which activated the expression of genes involved in the transport of carbohydrates, amino acids, and lipids in the funiculus.

### The expression of genes involved in sucrose-starch metabolism

**Fig. 7:**
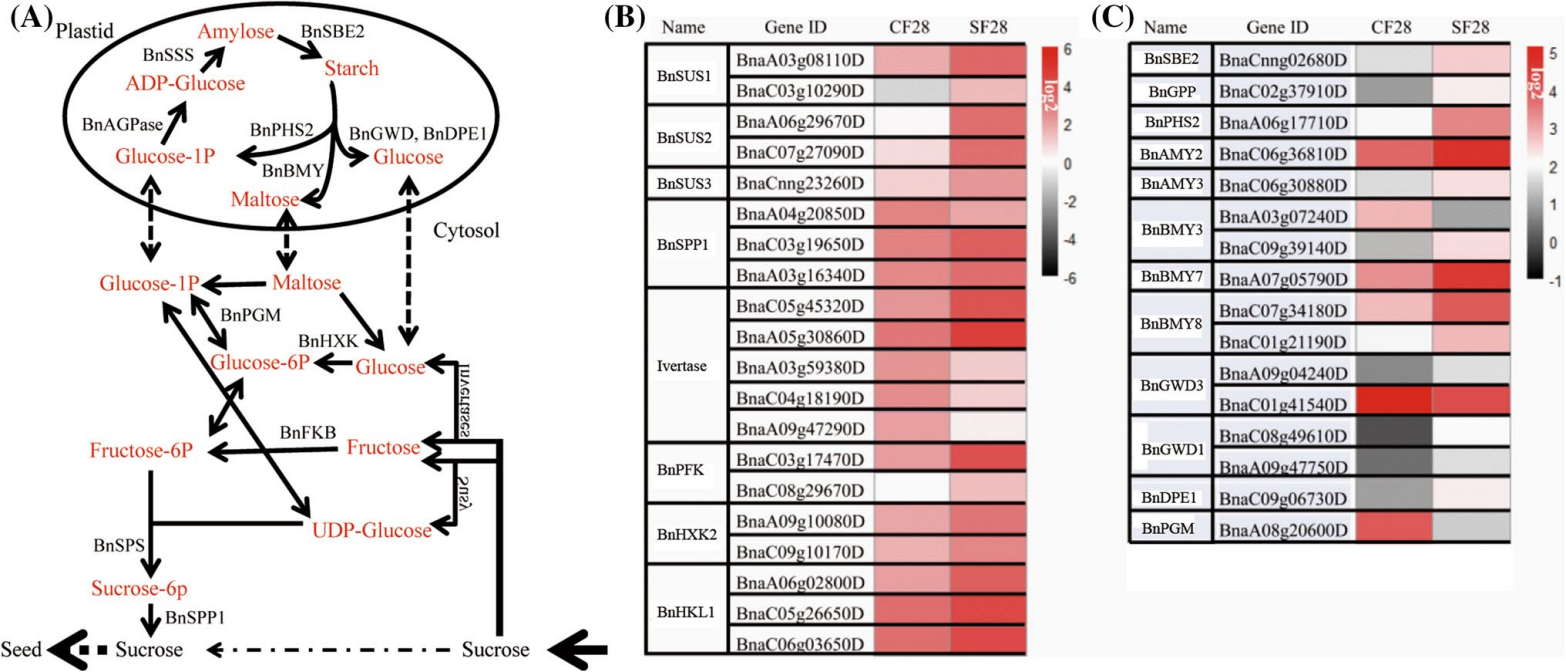
The expression of sucrose-starch metabolism genes in CF28 and SF28. **a** Schematic model of sucrose-starch metabolism in the funiculus. **b** The genes encoding sucrose metabolism enzymes. **c** The genes encoding starch metabolism enzymes

Sucrose in the funiculus is imported from the silique wall and a large part of this sucrose transfers to seeds, while the remainder could be degraded by metabolic enzymes in the funiculus (Fig. 7a). We analyzed the genes involved in sucrose metabolism in SF28 and found that the expression of a subset of genes significantly changed compared with control, non-shaded funiculi. Invertase and SUS (sucrose synthase) cleave sucrose to produce glucose, fructose, and UDP-glucose (Fig. 7a, b). The transcripts encoding BnSUS1, BnSUS2, and BnSUS3, were significantly upregulated in SF28, and three out of five transcripts encoding invertase were also upregulated (Fig. 7b). These enzymes produce monosaccharides, which could be metabolized to glucose-1p by BnHXK (hexokinase) and BnPGM (phosphoglucomutase); the *BnHXK* and *BnPGM* transcripts were also upregulated in SF28 (Fig. 7a, b). Subsequently, glucose-1p could be imported into plastids to synthesize starch by a series of enzymes including BnAGPase (ADP-glucose pyrophosphorylase), BnSSS (soluble starch synthase) and BnSBE2 (starch branching enzyme 2) (Fig. 7a, c). The transcripts encoding the enzymes BnAGPase and BnSBE2 were upregulated in SF28 (Fig. 7c). However, the transcript encoding BnSSS, the key enzyme for starch synthesis, was unchanged in SF28 compared with CF28, suggested a distinct regulation for *BnSSS*.

Interestingly, the transcript levels of genes encoding starch-degrading enzymes, such as BnPHS2 (glucan phosphorylase), BnBMY (beta-amylase), BnGWD (glucan water dikinase), and BnDPE1 (disproportionating enzyme 1), also were markedly upregulated in SF28 (Fig. 7a, c), indicating that the degradation of starch increased in SF28. The products of starch degradation, glucose-1p, maltose, and glucose, could be exported to the cytosol for glycolysis or sucrose synthesis (Fig. 7a). The mRNA abundances of sucrose synthesis enzymes in cytosol, BnSPS (sucrose phosphate synthase 1) and BnSPP1 (sucrose-phosphatase 1), were markedly higher in SF28 (Fig. 7b), although the contents of sucrose were lower in SF28 than in CF28 (Fig. 5d).

### Expression of genes involved in lipid metabolism in the funiculus

The synthesis of fatty acids (FA) and triacylglycerols (TAG) occurs primarily in the embryo and other zygotic tissues of the seed (Focks and Benning 1998; Baud and Lepiniec 2009); however, our dynamic metabolite profiling of the funiculus detected 23 fatty acids and lipids and showed that their contents increased during seed maturation, but drastically decreased in SF28. In the transcriptome, we found 151 mRNAs encoding enzymes associated with a variety of biological processes including lipid synthesis, transfer, storage, and degradation; their transcript levels also changed in SF28 compared with CF28 (Supplementary Table S3). Plants synthesize FA *de novo* mainly in plastids by a series of enzymes, including the FA synthase (FAS) multi-enzyme complex. We found that fifteen out of the sixteen (94 %) transcripts encoding the subunits of FAS showed upregulated expression in SF28, except BnaC07g17300D, encoding the enzyme 3-ketoacyl-CoA synthase 20, which decreased up to threefold in SF28 compared with CF28 (Supplementary Table S3). The expression of seven genes encoding fatty acid desaturase (FAD), including *BnFAD2, BnFAD5*, and *BnFAD7*, increased in SF28 (Supplementary Table S3). In contrast to this, thirteen FAs identified in the metabolite profile of SF28, including stearic acid, linolenic acid, and palmitic acid, were significantly reduced (Fig. 5e).

The abundance of 46 mRNAs encoding enzymes and proteins for synthesizing TAG, phospholipids, and glycolipids, and for lipid transfer and storage, showed distinct changes in SF28 (Supplementary Table S3). For example, 25 (54 %) of these mRNAs increased in abundance, including those encoding lipid transfer protein 2 (BnaA04g22070D), phosphatidylserine decarboxylase 1 (BnaAnng13400D), and CDP-diacylglycerol synthase 1. TAG synthesis is initiated in the endoplasmic reticulum by glycerol-3-phosphate acyltransferase and the reaction is sequentially completed by lysophosphatidic acid acyltransferase and diacylglycerol acyltransferase (Slabas and Fawcett 1992; Ohlrogge and Browse 1995; Voelker and Kinney 2001). Five mRNAs encoding glycerol-3-phosphate acyltransferase (BnaCnng08770D, BnaA10g00370D, BnaC03g34280D, BnaC05g00450D, BnaC09g49330D, BnaC09g49330D), showed reduced expression in SF28 (Supplementary Table S3). Consistent with this, the contents of 2-monostearin and glycerol monostearate were reduced in SF28. However, the abundance of mRNAs encoding lysophosphatidic acid acyltransferase (BnaA02g17090D) and diacylglycerol acyltransferase (BnaCnng43880D, BnaA09g24560D) increased in SF28 (Supplementary Table S3). These results showed that the genes encoding enzymes for TAG assembly are regulated by different mechanisms. Moreover, transcript abundance of the oleosin genes, which encode proteins forming the oil bodies for storage of TAG, also increased in SF28. Moreover, 59 transcripts involved in lipid degradation, including phospholipids, glycolipids and TAG, also obviously changed in SF28, 39 (66 %) with upregulated expression (Supplementary Table S3). For instance, the expression of the gene encoding lysophospholipase 2, an enzyme that degrades TAG, increased in SF28.

### Expression of genes involved in amino acid metabolism in the funiculus

Amino acids are one of main nutritional constituents of the funiculus and some amino acids showed close correlations with seed fresh weight (Supplementary Table S1). Moreover, the contents of most amino acids (except l-valine) decreased in SF28 compared with CF28 (Fig. 5f). To explore amino acid metabolic genes, we conducted pathway analysis, which found 145 genes involved in amino acid metabolism in the funiculus (Supplementary Table S4), including 40 involved in amino acid degradation and 105 involved in amino acid synthesis. Surprisingly, the abundances of 101 (70 %) genes increased in SF28 compared to CF28 (Supplementary Table S4), indicating that the expression of these genes was negatively regulated by the amino acid contents. The upregulated group included 68 genes associated with amino acid synthesis, such as tryptophan synthase (BnaA10g09090D, BnaC09g31260D, BnaC08g25400D, BnaA09g34470D), aspartate aminotransferase (BnaA03g51850D, BnaC03g04830D, BnaC04g41630D, BnaA03g03400D), and GABA transaminase (BnaA05g17430D, BnaC06g27330D, BnaA07g25530D). However, the genes encoding tryptophan degradation enzymes, beta-hydroxyisobutyryl-CoA hydrolase 1 (BnaA02g34880D, BnaC02g45100D), and 3-hydroxyacyl-CoA dehydratase 1 (BnaC08g09570D), were also upregulated in SF28, indicating that the synthesis and degradation of amino acids is a dynamic balance in the funiculus.

## Discussion

The funiculus is the sole communicational channel between the seed and the maternal plant (Belmonte et al. 2013). The development and synthesis of storage reserves in seeds require numerous nutrients imported from the maternal photosynthetic tissues via the funiculus. The nutrient status and the activity of metabolic pathways in the funiculus regulate the nutrition supplies for the seed, and thus affect the quality and quantity of the storage reserves in the seed (Millar et al. 2015). In this study, we measured the dynamic metabolite profiles in the funiculus during seed maturation in *B. napus* and identified important metabolites that correlated with seed fresh weight. Some of these metabolites in the funiculus decreased when we blocked photosynthesis in the silique wall with opaque aluminum foil; moreover, we observed changes in expression of a subset of genes involved in nutrient transport and metabolism of sugar, lipids, and amino acids. These results demonstrate that the contents of metabolites and the activities of their metabolic pathways in the funiculus have important implications for seed filling. These insights also improve our understanding of the function of the funiculus in controlling seed development, particularly the accumulation of storage reserves in the seed.

### Nutrients in the funiculus correlated closely with seed filling

The funiculus is essential for seed development (Chan and Belmonte 2013; Millar et al. 2015). Our data on the increase in the length of funiculus from 7 to 21 DAF (Fig. 1g) showed that the funiculus was ready to service the seed at a structural level. Previous histology studies revealed that the funiculi of *B. napus* (Chan and Belmonte 2013; Millar et al. 2015), Arabidopsis (Belmonte et al. 2013; Khan et al. 2015), and *Phaseolus vulgaris* (Mawson et al. 1994) showed proliferation of the vasculature tissue from the ovule to the globular stage, supporting transport as a primary function during seed filling. During the development of *B. napus* seed, the seed fresh weight increased nearly twofold during the morphogenesis phase from 7 to 21 DAF, and then continuously increased during seed filling from 21 to 35 DAF (Fig. 1i). With the seed weight increase, the contents of nutrients in the funiculus, including carbohydrates, amino acids, and fatty acids and lipids, increased from 21 to 35 DAF. Given that the growth of the funiculus mostly stopped at 21 DAF (Fig. 1g), theoretically, the funiculus did not need a lot of nutrients to maintain its physical activity after this time. However the levels of nutrients showed a continual, rapid increase (Fig. 2b), indicating that these nutrients were destined for seed filling. So the pattern of these metabolites likely reflects nutrient demand during seed maturation and our correlation analysis between metabolites and seed fresh weight also indicated these metabolites are important for seed filling (Supplemental Table S2).

Sucrose is the major form of carbohydrate for long-distance transport (Housley et al. 1977; Riesmeier et al. 1994) and sucrose levels rapidly increased from 21 to 35 DAF in the funiculus (Fig. 3b), suggesting that seeds have an extremely strong demand for sucrose during seed filling. Sucrose may be directly transported into seeds by sucrose transporters (SUT) (Weise et al. 2000; Sauer 2007), without needing to be turned over into other forms, resulting in a linear correlation between sucrose and seed weight. A previous microarray study showed that *AtSUT4* is highly expressed in the Arabidopsis funiculus (Khan et al. 2015) and in our transcriptome data, *BnSUT1, BnSUT4, BnSUT5, BnSUT6*, and other carbohydrate transporter genes *BnGTP1, BnUTR2, BnPMT5, BnVGT1, BnPLT5* and *BnPLT2* were also active in the *B. napus* funiculus (Fig. 6a). These activated transporters reflect the diversity of carbohydrate transport in the *B. napus* funiculus. Consistent with this, funiculus metabolite profiles showed multiple monosaccharides closely correlated with seed filling (Supplemental Table S2).

Beside the carbohydrate-related genes, a subset of genes involved in transport of amino acids and fatty acids and lipid were active in the funiculus (Fig. 6b, c); as a result a large number of amino acids, and fatty acids would likely be transported into the seed. Our previous profiling of seed metabolites also showed the contents of some amino acids and fatty acids increased during seed maturation (Tan et al. 2015). Thus, our profiles of metabolites and transcripts revealed a strong indication of the function of the funiculus in nutrient transfer, although we cannot provide direct evidence using metabolic flow analysis of isotope labeling to prove these metabolites were transported to the seed.

### Shading the silique significantly reduced nutrient levels in the silique wall of *B. napus*

In *B. napus*, the seed is the most prominent sink organ (Diepenbrock 2000; Jullien et al. 2011). At early stages at which the silique is not fully developed, carbon compounds are transported to the seed through the phloem network of the stem and silique wall. These compounds mainly come from leaf photosynthesis. When the seed proceeds to the filling stage, however, most carbon compounds come from the silique wall, although some nutrients translocate to the seed from the leaf and green stem. This mainly occurs because the leaf has initiated senescence, resulting in a dramatic decline in leaf photosynthesis during seed filling (Pechan and Morgan 1985; Hua et al. 2012; Tan et al. 2015). Unlike many starch-storing leaves of C_3_ dicotyledonous species, the canola silique wall preferentially partitions photosynthate into sucrose (King et al. 1997). A portion of this sucrose appears to be hydrolyzed by acid invertase into hexose for vacuolar storage and for internal metabolic events. Unlike the source leaf, the silique wall carbohydrates were rapidly translocated to the seed through the phloem; the nutrients synthesized in the silique wall showed a marked diurnal increase and little was used at night (King et al. 1997).

To investigate the nutrient relationship between the silique wall and funiculus during seed filling, we blocked photosynthesis of the silique by shading it with aluminum foil, and thus reducing the nutrient level of the silique wall. The inflorescence of *B. napus* is racemose and flowers from the bottom to the top; therefore it is difficult to find siliques of the same developmental stages. To obtain the precise, non-shaded control, we wrapped half of the silique with aluminum foil and left the other half of the silique as the non-shaded control. Theoretically, blocking the photosynthesis of the shaded part of the silique (SSW) should cause its nutrient level to rapidly decrease compared with the non-shaded part (CSW). However, the nutrient level did not completely decrease in the silique wall shaded with aluminum foil after 7 days (Fig. 4b). The levels of carbohydrates and other complex metabolites decreased slightly in SSW compared to CSW, but the levels of amino acids and their derivatives, and organic acids were reduced by 72.4 and 33.1 %, respectively (Fig. 4b). This suggested that some nutrient supply partly compensated for the decrease caused by blocking silique photosynthesis. There are three possible ways to compensate for the decrease in nutrients in the shaded silique wall. First, the large nutrient molecules stored in the silique wall cell, such as starch and protein, could be metabolized to increase the nutrient level. Second, the leaf and stem could translocate some nutrients to the silique wall through the silique phloem. Third, the adjacent, non-shaded part of the silique could provide photoassimilate to the shaded part of the silique. In summary, during seed filling, the photoassimilate of the silique provides a large amount of nutrition for the silique wall and shading the silique significantly reduced the nutrient level although the leaf, stem, and non-shaded silique part could transport small amounts of nutrient through the silique phloem.

### The funiculus functions in transient storage of nutrients

The nutrients imported from the silique wall into the funiculus will further transfer to the seed via the phloem (Millar et al. 2015), and the transporters specific to each nutrient determine the ratio of nutrients that move to the seed (Weber et al. 1997). At early developmental stages, the funiculus is transparent and during development (Fig. 1a, b), the funiculus turns green as the cells develop functional chloroplasts during seed filling (Fig. 1c–d, Khan et al. 2015). However, due to the low levels of light and CO_2_, the chloroplasts in the funiculus likely receive little light energy and thus cannot synthesize a large quantity of starch, like the chloroplasts in the seed embryo (Asokanthan et al. 1997; Ruuska et al. 2004). However, ultrastructural analysis revealed that chloroplasts of the funiculus do contain starch grains (Chan and Belmonte 2013; Khan et al. 2015). Our transcriptome data indicated the starch could be synthesized by plastids using the degradation products of sucrose imported from the silique wall, as the genes associated with sucrose degradation and starch synthesis were active in the funiculus (Fig. 7a, c).

A previous study revealed that the funiculus plays a role as a center for fatty acid biosynthesis, expressing a subset of genes involved fatty acid synthesis (Khan et al. 2015). Our study also supports this idea. In the metabolite profiles of the funiculus, we identified 23 fatty acids and glycerol monostearates (Supplemental Table S1), and the transcriptome data showed activation of the genes involved in not only fatty acid pathway, but also in the fatty acid modification pathway and TAG assembly and storage pathways (Supplemental Table S3).

In fact, the funiculus cannot store large quantities of nutrients for the long-term, so the synthesized starch and TAG were likely transiently stored. Once the nutrient content decreased in funiculus, the starch and TAG would be degraded and transported to the seed. Our data revealed that when the levels of sucrose, FA, and amino acids in the SF28 metabolite profile declined (Fig. 5d–e), this resulting in increased expression of genes involved in starch and TAG catabolism in SF28 (Fig. 7; Supplemental Table S3).

### Nutrient levels negatively regulate gene expression in the funiculus

The transcriptome of the Arabidopsis funiculus has a number of gene networks hypothesized to be responsible for key biological processes (Khan et al. 2015). In this study, our analysis of metabolite and transcript levels found these predicted biological processes also occur in the *B. napus* funiculus, particularly the processes involving primary metabolites, which revealed that the funiculus acts as a center for nutrient turnover and management of nutrient transfer.

In addition, plant gene responses to changing metabolite status can vary markedly, particularly for the genes involved in metabolism, which are often regulated by their products (Koch 1996; Hermans et al. 2006). Our transcriptome analysis showed that the genes associated with various metabolic pathways were activated mainly by reduction of the levels of metabolites, such as the amino acid (Supplemental Table S4), sucrose, and fatty acid pathways (Fig. 7b, c; Supplemental Table S3), and especially the genes for nutrient transfer (Fig. 6). In terms of the major role of the funiculus, this negative regulatory mechanism can help provide more nutrients for seed development, thus potentially improving the reproductive success of the plant.

## Conclusions

In *B. napus*, the funiculus is an important, yet simple structure connecting the maternal plant and the developing seed. Metabolite profiling of the funiculus identified large sets of metabolites dynamically correlated with seed filling during seed maturation. Transcripts encoding proteins involved in a number of biological processes including metabolite transport, sucrose-starch metabolism, FA and TAG metabolism, and amino acid turnover were identified by transcriptome analysis. Furthermore, comparison of metabolites and transcript levels in shaded siliques revealed key metabolic and transcriptional changes resulting from decreased photosynthesis in this key tissue that connects the seed with the rest of the plant. These results enhance our understanding of the functions of the funiculus in seed development and nutrition storage. The identification of key metabolites and the genes associated with the transport and turnover of these metabolites could be used to inform strategies to improve the quantity and quality of *B. napus* seed.

## Electronic supplementary material

Below is the link to the electronic supplementary material.

Supplemental Table S1. The dynamic metabolite profiles in funiculus at three development stage of seed maturation. (XLS 111 KB)

Supplemental Table S2. The metabolites that show close correlation with the seed fresh weight in the funiculus. (XLS 50 KB)

Supplemental Table S3. The genes involved in lipid metabolism identified in funiculus transcriptome. (XLS 67 KB)

Supplemental Table S4. The genes involved in amino acid metabolism identified in funiculus transcriptome. (XLS 79 KB)
